# Fabrication of an Au-doped Cu/Fe oxide-polymer core–shell nanoreactor with chemodynamic and photodynamic dual effects as potential cancer therapeutic agents

**DOI:** 10.1038/s41598-022-23002-5

**Published:** 2022-11-04

**Authors:** Chun-Kai Sun, Yin-Hsu Wang, Yu-Liang Chen, Ting-Yu Lu, Hsi-Ying Chen, Shih-Chin Pan, Po-Chun Chen, Mei-Yi Liao, Jiashing Yu

**Affiliations:** 1 Department of Chemical Engineering, National Taiwan University, Taipei, 10617 Taiwan; 2 Materials Science and Engineering Program, University of California San Diego, La Jolla, CA 92093 USA; 3 Department of Materials and Mineral Resources Engineering, Institute of Materials Science and Engineering, National Taipei University of Technology, Taipei, 10608 Taiwan; 4 Department of Applied Chemistry, National Pingtung University, Pingtung, 90003 Taiwan

**Keywords:** Biotechnology, Cancer, Chemistry, Engineering, Materials science, Nanoscience and technology

## Abstract

Nanoparticles are widely used in biomedical applications and cancer treatments due to their minute scale, multi-function, and long retention time. Among the various nanoparticles, the unique optical property derived from the localized surface plasmon resonance effect of metallic nanoparticles is a primary reason that metallic nanoparticles are researched and applied. Copper and Iron nanoparticles have the potential to generate hydroxyl radicals in excess H_2_O_2_ via Fenton or Fenton-like reactions. On the other hand, gold nanoparticles equipped with a photosensitizer can transfer the energy of photons to chemical energy and enhance the production of singlet oxygen, which is suitable for cancer treatment. With the actions of these two reactive oxygen species in the tumor microenvironment, cell apoptosis can further be induced. In this work, we first synthesized dual metal nanoparticles with poly[styrene-alt-(maleic acid, sodium salt)(Cu ferrite oxide-polymer) by a simple one-step hydrothermal reduction reaction. Then, gold(III) was reduced and doped into the structure, which formed a triple metal structure, Au-doped Cu ferrite nanoparticles (Au/Cu ferrite oxide-polymer NPs). The metal ratio of the product could be controlled by manipulating the Fe/Cu ratio of reactants and the sequence of addition of reactants. The core–shell structure was verified by transmission electron microscopy. Moreover, the hydroxyl radical and singlet oxygen generation ability of Au/Cu ferrite oxide-polymer was proved. The chemodynamic and photodynamic effect was measured, and the in vitro ROS generation was observed. Furthermore, the behavior of endocytosis by cancer cells could be controlled by the magnetic field. The result indicated that Au/Cu ferrite oxide-polymer core–shell nanoreactor is a potential agent for chemodynamic/photodynamic synergetic therapy.

## Introduction

The application of metallic nanoparticles is now a popular solution in various fields, including catalysis, semiconductors, as well as for biomedical uses^1–4^. The unique physiochemical properties of the high surface-volume ratio, localized surface plasmon resonance effect, and internalization, owing to the small size of nanoparticles, provide new perspectives for solving a variety of biomedical problems^5–10^.

Among the various applications of nanoparticles in the biomedical field, cancer treatment is a suitable subject. Due to the short outgrowth of tumors, several differences exist between tumor tissue and normal tissue, including low pH value, hypoxia microenvironment, and defective vessels^11,12^. The abnormal structure and function of tumor vessels with leaks and defects provide sites for nanoparticles to approach the tumor tissues and passively accumulate. This phenomenon is called the enhanced permeability and retention effect^13^.

Photodynamic therapy (PDT), an innovative and promising therapeutic modality for treating cancers, involves light, photosensitizers, and oxygen around the tissue^14,15^. Among them, photosensitizers essentially function as catalysts that can be activated by light of a specific wavelength and transfer the energy to surrounding oxygen molecules, resulting in the generation of reactive oxygen species (ROS) via Type I and Type II reactions^16–18^. With light absorption, these specific photosensitizers can undergo an intersystem crossing and further react in two ways: with biomolecules to generate ROS indirectly (Type I reaction) or directly with oxygen, which creates an energy transfer and results in singlet oxygen (^1^O_2_) generation (Type II reaction)^18^. Between them, ^1^O_2_ is extremely electrophilic and can directly oxidize electron-rich double bonds in biological molecules and macromolecules. It is believed to be the prime cytotoxic agent related to PDT^19^.

Gold nanoparticles have been extensively studied within biomedicine because of their photochemical, photophysical, and optical properties^20–22^. In particular, these have been widely used to deliver photosensitizer agents for PDT^16,23–25^. Gold nanoparticles are also found to enhance the singlet oxygen generation rate^26,27^. In addition, the biocompatibility and low toxicity of gold nanoparticles make it ideal for biomedical applications^20,28^. Methylene blue is one of the appropriate photosensitizers commonly used in photodynamic therapy due to its outstanding photochemical properties^19,29^. It has been reported that the monomer species of methylene blue favors the type II pathway^30^.

Chemodynamic therapy is another way to trigger cancer-cell apoptosis. Ferrous ion (Fe^2+^) and cuprous ion (Cu^+^) can catalyze H_2_O_2_ to generate highly cytotoxic hydroxyl radicals (·OH) via Fenton and Fenton-like reactions, respectively when tailored to the specific tumor microenvironment, which involves acidity and the overproduction of hydrogen peroxide (H_2_O_2_)^31–34^. Among the subtypes of ROS, ·OH has relatively high reactivity^35^. For this purpose, iron, and copper nanoparticles are two indispensable elements responsible for ROS generation.

Iron nanoparticles are widely used in biomedical applications due to their low toxicity, superparamagnetic property, and imaging ability. Moreover, H_2_O_2_ disproportion can be boosted in the existence of ionized Fe to generate ROS ad even produce oxygen in a high H_2_O_2_ environment by the Fenton reaction^36–39^. The biodegradability of Cu is also more significant than noble metals frequently used in cancer therapy, which can prevent accumulation in a living body^40–42^. It has been studied that the combination of iron and copper nanoparticles can synergically produce ROS, and the enhanced ROS generation ability has been proved^43–46^.

Although alloy nanoparticles have been widely studied, little research has been done to study the synthesis and application of copper-iron-gold triple metal nanostructures. The potential of copper, iron, and gold has already shown promise in the literature.

**Figure 1:**
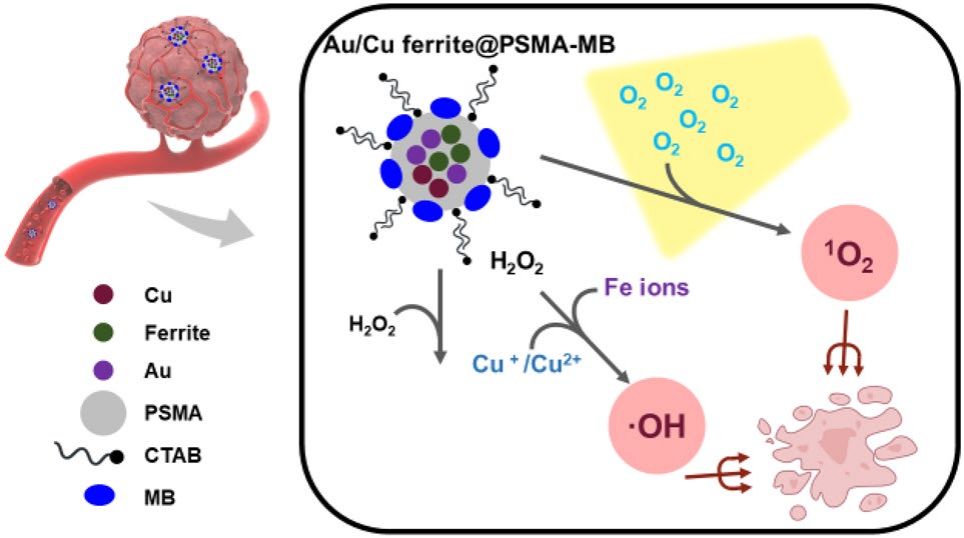
The mechanism of HeLa cell apoptosis is triggered by MB Au/Cu ferrite oxide-polymer core–shell NPs.

To understand in vitro ROS generation contributed by chemodynamic or photodynamic effects, an evaluation of cell activity before and after light irradiation was carried out. The pathways of HeLa cell apoptosis triggered by MB-Au/CuFe nanoparticles are illustrated in Fig. 1.

In previous studies, a wide variety of metal-core-polymer-shell nanoparticles with a core of Au, Cu, and Fe_3_O_4_ have been produced using one-step hydrothermal synthesis.^47^ Moreover, gold(III) derivatives were widely used to prepare gold nanoparticles via the chemical reduction method.^30^ In the research into Au polymer core–shell nanoparticles, the photodynamic effect of an Au photosensitizer composite was applied to generate reactive oxygen species (ROS). Besides gold nanoparticles, it was reported that Cu ferrite nanoparticles could enhance ROS generation by disproportionate reactions of H_2_O_2_.^43,44^ If a combination of gold and Cu Ferrite nanoparticles could be realized, the therapeutic effects of ROS-mediated therapy, including chemodynamic effect and photodynamic effect, could synergistically trigger in vitro ROS generation and thus enhance the treatment, leading to cell apoptosis. A two-step reaction, including hydrothermal synthesis and a chemical reduction method, is a possible approach to producing triple-metal nanoparticles. In this study, we designed the triple-metal nanoparticles loaded with a photosensitizer. CuFe oxide-polymer NP products with different Fe/Cu ratios were used in the Au/CuFe oxide-polymer NP synthesis. The synthesized nanoparticles were chemically and physically characterized. The photodynamic and chemodynamic ability were elucidated with singlet oxygen generation, hydroxyl radical formation, and in vitro ROS generation. The aim of this study is the clarify the role and mechanism played by the individual component of the synthesized nanoparticles and provide information for the future design of the metal-based nanoparticles in the biological application and possible therapeutic treatments.

## Results and discussion

After the synthesis and purification process, the Au/Cu ferrite nanoparticles (herein from now on/CuFe NPs) were well dispersed in DI water. In this study, each group's copper, iron, and gold concentration was quantified by atomic absorption (Table 1). For the CuFe NPs, the Fe/Cu ratio of the reactant was manipulated by the different amounts of Fe ion, while the amount of Cu ion and reductant (N_2_H_4_) was constant. Thus, the decrease of Cu concentration could have contributed to the increased Fe iron that competed with the reduction reaction of Cu ion. For the Au/CuFe NPs, the first process led to a higher Au concentration than the second one, which could be ascribed to the overall reaction time of two reducing agents, the Au/CuFe NPs and ascorbic acid (Vitamin C). Vitamin C did not participate in the Au doping reaction in the first 10 min for the second process, which weakened the ability to reduce hydrogen tetrachloroaurate(III) (HAuCl_4_). Furthermore, the Cu concentration was fixed for the Au doping reaction, so the CuFe(1:4) NPs contained more metal NPs than CuFe(4:1) NPs, resulting in a more significant amount of AuNPs reduced from HAuCl_4_. However, there was little copper left in the Au/CuFe NPs after the Au doping reaction since Cu is more inclined to ionize and dominate the redox reaction between the CuFe NPs and HAuCl_4_ compared to ferrite. At the same time, the concentration of Cu was still higher for the first process and the Au/CuFe(4:1) NPs, compared to the second one and the Au/CuFe(1:4) NPs, respectively. In addition, the CuFe(1:4) NPs gave rise to higher Fe concentrations in the Au/CuFe NPs than CuFe(4:1) NPs, and there was more Fe left in the Au/CuFe(1:4) NPs after the Au doping reaction than the Au/CuFe(4:1) NPs.

**Table 1:** Metal concentration of Au/Cu ferrite oxide-polymer core–shell NPs. (n = 6).

Types of Au/Cu ferrite oxide-polymer NPs	Metal Concentration		
	Cu (ppm)	Fe (ppm)	Au (ppm)
Cu ferrite(4:1)	52.00 $\pm$ 0.00	9.76 $\pm$ 0.29	–
Au(f)/Cu ferrite(4:1)	7.06 $\pm$ 2.56	3.46 $\pm$ 0.88	309.74 $\pm$ 22.81
Au(s)/Cu ferrite(4:1)	3.48 $\pm$ 0.30	7.77 $\pm$ 2.14	256.08 $\pm$ 10.13
Cu ferrite(1:4)	52.00 $\pm$ 0.00	238.28 $\pm$ 26.44	–
Au(f)/Cu ferrite(1:4)	6.23 $\pm$ 2.57	70.20 $\pm$ 20.23	325.25 $\pm$ 27.95
Au(s)/Cu ferrite(1:4)	2.08 $\pm$ 0.51	89.79 $\pm$ 17.29	280.11 $\pm$ 22.47

**Figure 2:**
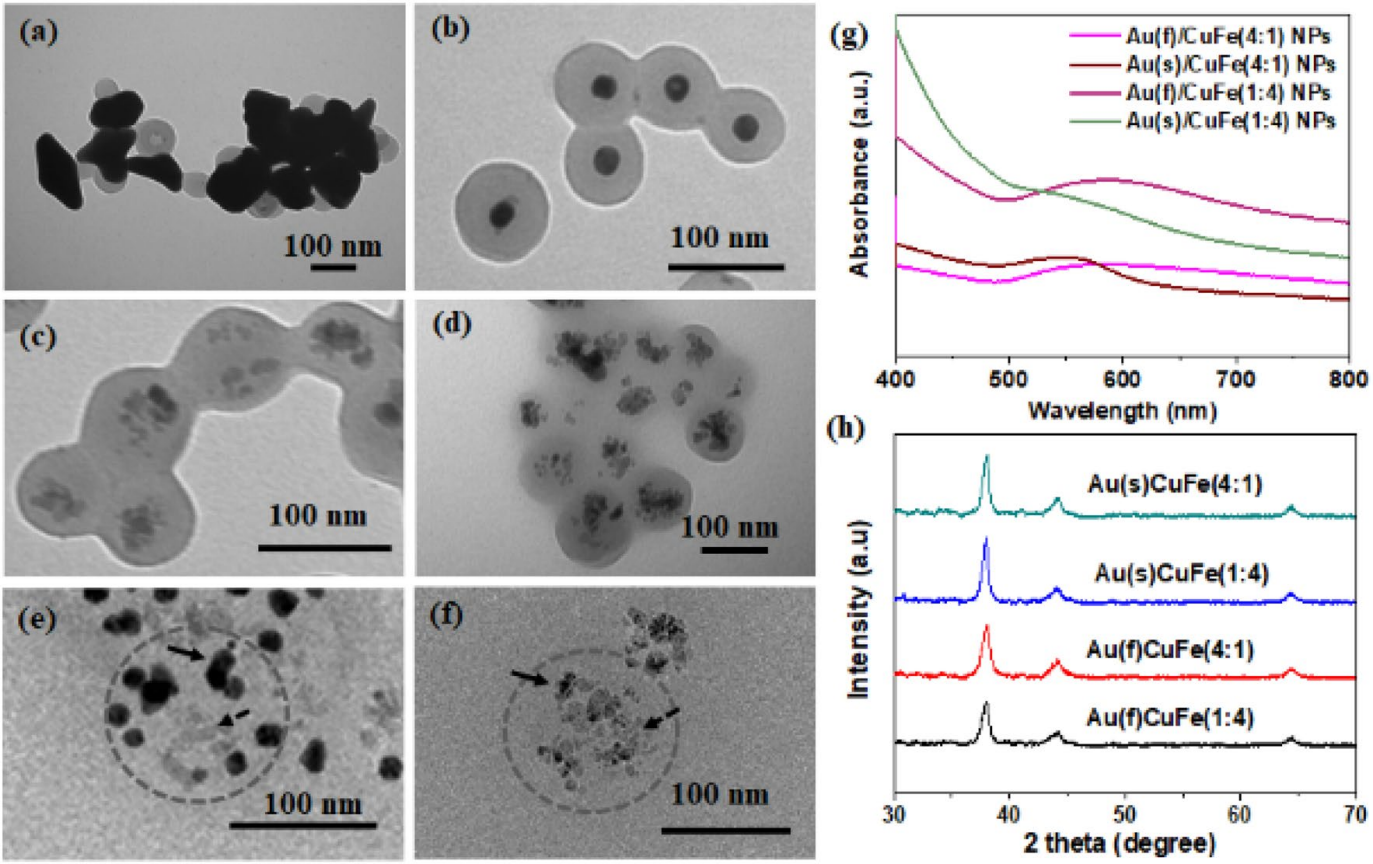
TEM images of the Au-doped Cu/Fe oxide-polymer nanoreactor synthesized with the different reaction parameters (**a**) Au(f)/CuFe(4:1), (**b**) Au(s)/CuFe(4:1), (**c**) Au(f)/CuFe(1:4), (**d**) Au(s)/CuFe(1:4), ), (e) HRTEM images of Au(f)/CuFe(1:4), (**f**) HRTEM images of Au(s)/CuFe(1:4) (**g**) UV–vis spectra, (h) XRD pattern.

Figure 2 shows transmission electron microscopy (TEM) images of Au-doped Cu/Fe oxide-polymer nanoreactors prepared with different synthesis parameters (Fig. 2a-d). The structures were confirmed with TEM images. The dark region, representing metal cores, is encapsulated in the light region, representing PSMA. In Fig. 2a-b, for the Au(f)/CuFe(4:1) NPs, the angular AuNPs were formed. For the Au(s)/CuFe(4:1) NPs, the single and round-shaped cores of AuNPs were produced. In Fig. 2c-d TEM images and Fig. 2e,f HRTEM images, the averagely-doped AuNPs were observed, evenly distributed with Cu and ferrite NPs to form multiple-core structures.

Figure 2g shows the UV–vis spectra of the Au-doped Cu/Fe oxide-polymer nanoreactor. The primary absorption band at ~ 540–580 nm was attributed to the typical SPR property of Au nanostructures in the Au-doped Cu/Fe oxide-polymer nanoreactor. The Au(f)/CuFe NP shows a broad absorption bandwidth after 500 nm, while the Au(s)/CuFe NP shows a relatively narrow one. Additionally, after HCl corrosion, the remaining NPs in the cores were supposed to be AuNPs. In Fig. 2e, Au(f)/CuFe(4:1) NPs transformed into multiple round-shaped and relatively small particles, inferred to be AuNPs. In Fig. S1, a portion of the metal core was removed by HCl, deduced to be Cu and ferrite NPs. In Fig. S1a–d, the cores were still occupied by widespread AuNPs after HCl corrosion, confirming that Au was successfully doped in the core of the nanoreactors. We can see that the absorption peaks contributed by the AuNPs still existed after corrosion by 0.05 M HCl (Fig. S2), which can be inferred that the AuNPs were not etched by the acid. As shown in Fig. 2h, the Au-doped Cu/Fe oxide-polymer nanoreactor in the product was not detected by X-ray diffraction (XRD). The fcc-structured Au material in the resulting crystal indicated that Fe was immobilized on the PSMA nanoparticles instead of releasing Fe ions to react with Au.

To characterize the behavior of Au/CuFe NPs in the aqueous phase, dynamic light scattering was applied to determine the hydrodynamic diameter and zeta potential. In Fig. S3, after the Au doping reaction, the surface charge changes from negative to positive due to the presence of CTAB in the shell of Au/CuFe NPs. To understand the stability of NPs, different Au/CuFe NPs were dispersed in PBS solvent with three different pH values, including 4.0 (acidic), 7.4 (neutral), and 10.0 (basic). The solution was centrifuged at different time intervals, and optical properties were measured (Fig. S4). In the PBS-NPs system, the optical change can be ascribed to salt-induced aggregation. For each group of Au/CuFe NPs, the gradual decrease of the absorption peak in intensity could be observed in pH 4.0 and 7.4 PBS; however, in pH 10.0 PBS, the absorption peaks were quickly weakened after 4 h quiescence.

**Figure 3:**
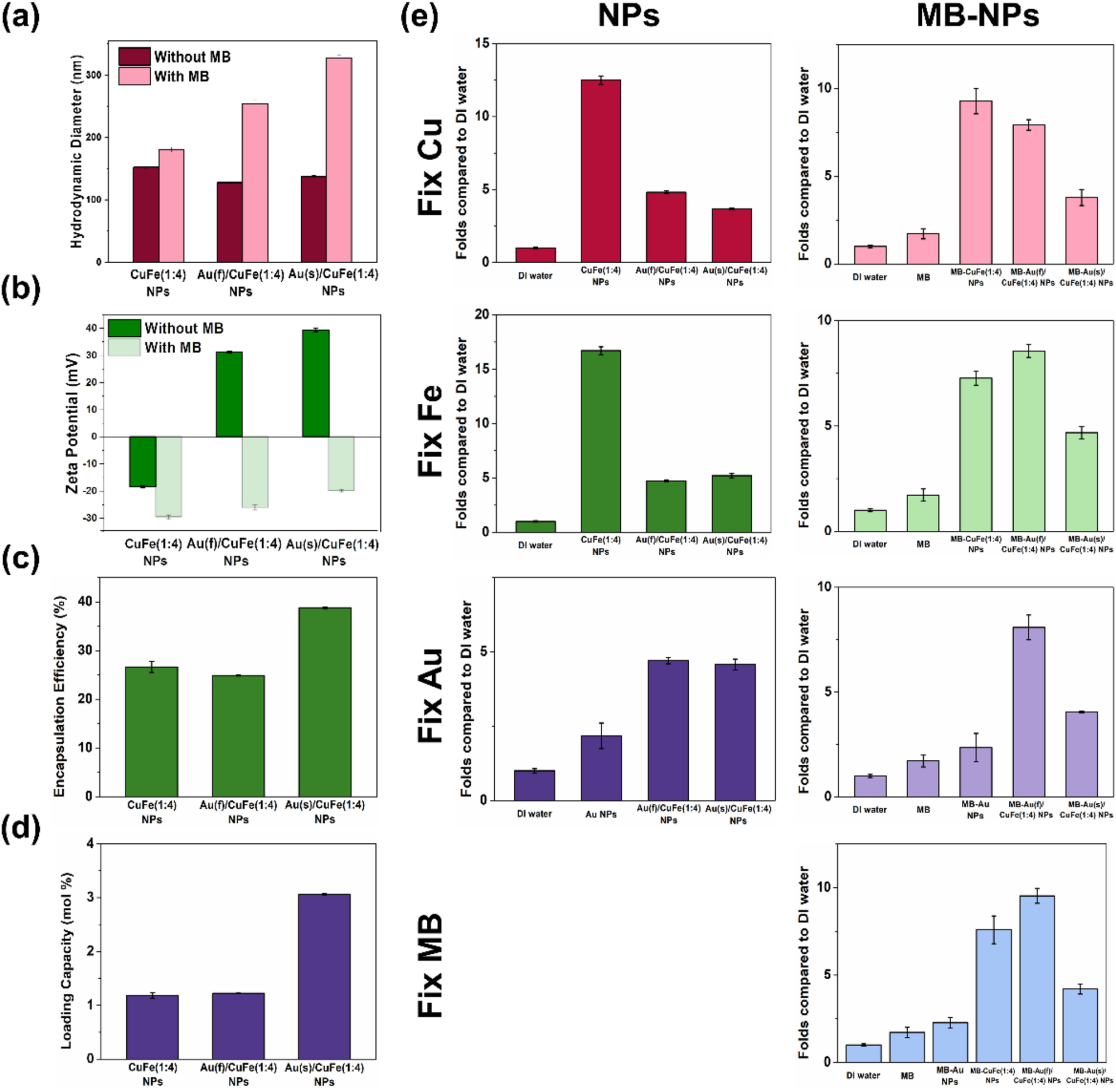
Characterization of MB-NPs. (**a**) Hydrodynamic diameter. (n = 3) (**b**) Zeta potential. (n = 3) (**c**) Encapsulation efficiency. (n = 3) (**d**) Loading capacity. (n = 3). (**e**) The ability of hydroxyl radical generation.

Based on the characterization test results and the NP-cell interaction performance, CuFe(4:1) NPs, Au(f)/CuFe(4:1) NPs, and Au(s)/CuFe(4:1) NPs were selected and introduced to present the ROS-mediated effect of Au/CuFe NPs. To undergo photodynamic ablation of cancer cells, methylene blue (MB) was applied as a photosensitizer and embedded between the layers of PSMA with the help of a pi-pi stacking interaction, which formed MB-immobilized CuFe NPs and Au/CuFe NPs (MB-CuFe NPs and MB- Au/CuFe NPs). The optical property was changed after MB loading and showed an absorption peak at around 660 nm for both Au(f)/CuFe(4:1) NPs and Au(s)/CuFe(4:1) NPs (Fig. S2), which confirmed the successful loading of MB. In Fig. 3a-b, the hydrodynamic size and surface charge are also changed. A larger structure was formed, and the nanocrystal was more negatively-charged on the surface, which was more obvious for the Au/CuFe NPs that were with the positively-charged surface before MB-immobilization. In Fig. 3c, two groups of Au/CuFe(1:4) NPs showed a similar EE% at around 25%, while CuFe(1:4) NPs showed about 39%. In Fig. 3d, two groups of Au/CuFe(1:4) NPs also showed a similar LD%, slightly lower than that of CuFe(1:4) NPs. The results were advantageous for the upcoming tests due to the relative total mass amount of Fenton catalysts, copper, and iron for the three groups as the MB concentration was fixed. MB-CuFe(4:1) NPs, MB-Au(f)/CuFe(4:1) NPs, and MB-Au(s)/CuFe(4:1) NPs were later diluted with DI water to 10 μM of MB concentration for further use, and the composition of three nanocrystals as shown in Table S1.

After RNO is added and mixed with the samples, the absorption peak at the wavelength of 440 nm was noticed (Fig. S7a). After 10 min of light irradiation, the reduction of the characteristic peak in intensity could also be observed and measured, implying the generation of singlet oxygen (^1^O_2_). In Fig. S7b–c, DI water showed almost no ^1^O_2_ generation, while MB showed a certain amount of ^1^O_2_ generated, set as 100%. In Fig. S7d–f, the three groups with the same concentration of MB showed the equally matched ability of ^1^O_2_ generation, which were all around 75% compared to MB only (Table S2); however, this was expected since MB was fixed in the assay.

To understand which component of the Au/CuFe NPs contributed to the ability of hydroxyl radical (·OH) generation, all the groups with and without immobilized MB underwent a TA test. The concentration of Cu, Fe, Au, and MB was fixed at 1 ppm, 20 ppm, 60 ppm, and 10 uM, respectively. The results were presented by TAOH-contributed fluorescent intensity folds compared to DI water only. (Fig. 3e) As the Cu and Fe concentrations were fixed, CuFe(1:4) NP had the highest ability of ·OH generation among non-MB-immobilized groups due to the highest Fenton catalyst concentration either when Cu or Fe was fixed. As Au concentration was fixed, Au(f)/CuFe(1:4) NPs and Au(s)/CuFe(1:4) NPs showed a similar ability of ·OH generation. However, the Au(f)/CuFe(1:4) NP showed the highest production of ·OH after MB immobilization, whether Fe or MB concentration was fixed.

**Figure 4:**
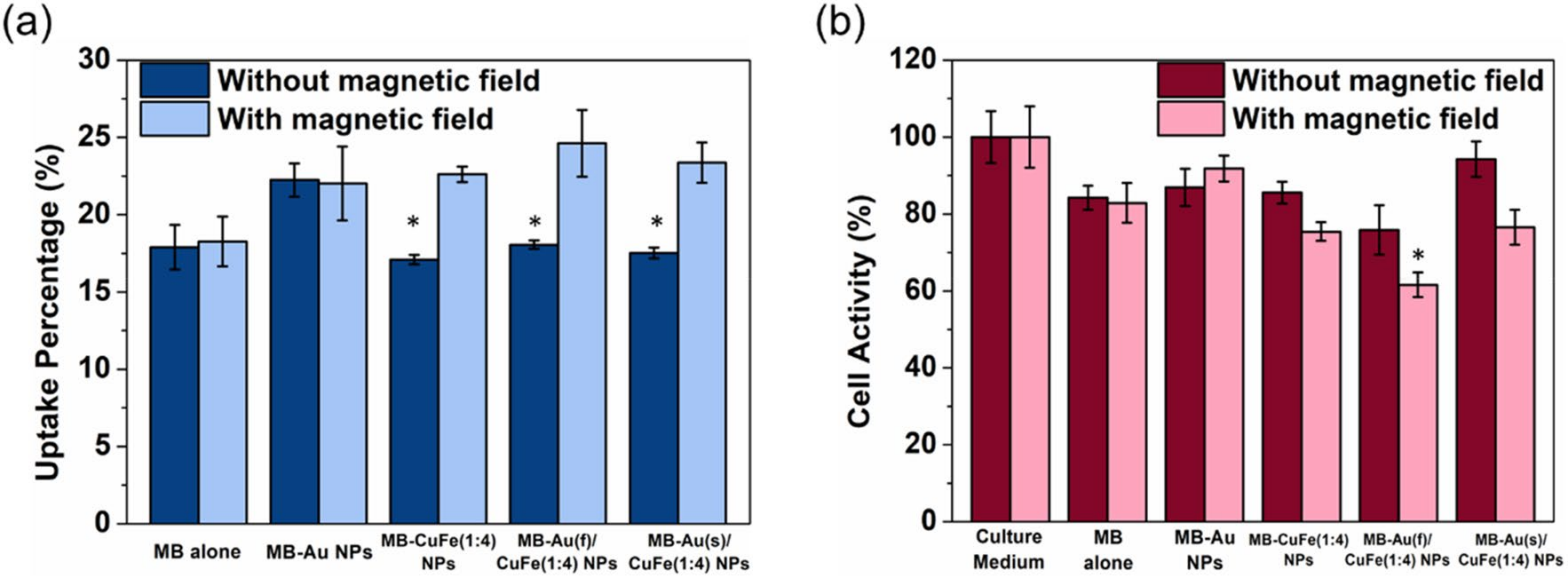
(**a**) Cellular uptake and (**b**) cell activity after 4 h incubation with and without a magnetic field. (n = 4).

After 4 h of incubation with MB-CuFe NPs or MB-Au/CuFe NPs, the uptake of MB was quantified (Fig. 4a). Furthermore, a magnetic field (MF) was applied to understand whether or not the behavior could be manipulated by magnetic force. Without a MF, three groups showed a similar uptake of MB at around 18%, and the values were lifted to an average of 24% with MF, thanks to the ferromagnetic property of ferrite. MB was qualified to be fixed for each group in the later cell experiments based on the result. The corresponding cell activity was also estimated (Fig. 4b). With a MF, the cell activity of all groups decreased, which verified the elevated uptake of MB with a MF. Among them, Au(f)/CuFe(1:4) NP showed the highest toxicity, with CuFe(1:4) NP next, and Au(s)/CuFe(1:4) NP at the lowest, which corroborates the result in the TA test, and the toxicity contributed by the chemodynamic effect was also confirmed.

**Figure 5:**
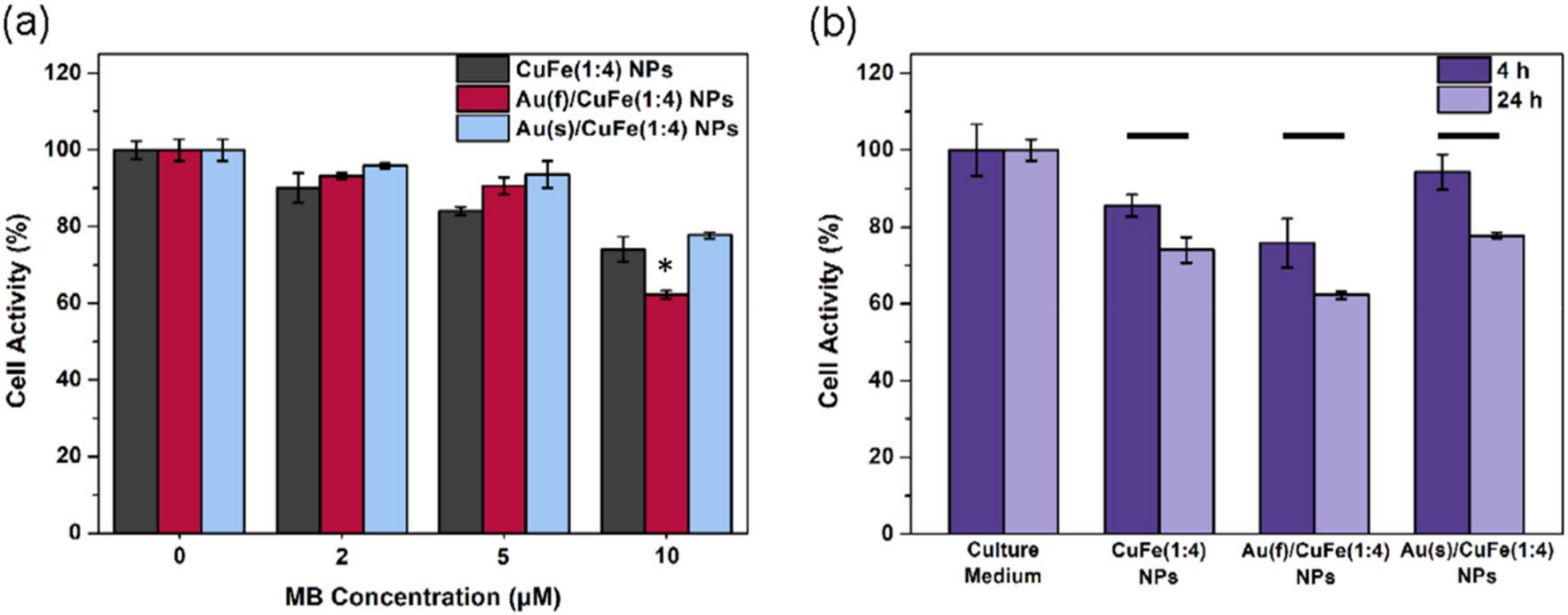
Dark toxicity of MB-NPs. (**a**) Cell activity after 24 h of incubation. (**b**) After 4 h and 24 h of incubation, cell activity with 10 μM MB of MB-NPs. (n = 4).

After 24 h of incubation with MB-CuFe NPs or MB-Au/CuFe NPs containing different MB concentrations, the dark cytotoxic activity was estimated. In Fig. 5a, at a low concentration of MB, MB-CuFe(1:4) NP shows the highest toxicity, while at a high concentration of MB, MB-Au/CuFe(1:4) NP becomes the most toxic to cells. Compared to the incubation time of 4 h, the cell activity also decreases due to the long-term action of the chemodynamic effect as the incubation time stretches to 24 h. (Fig. 5b).

**Figure 6:**
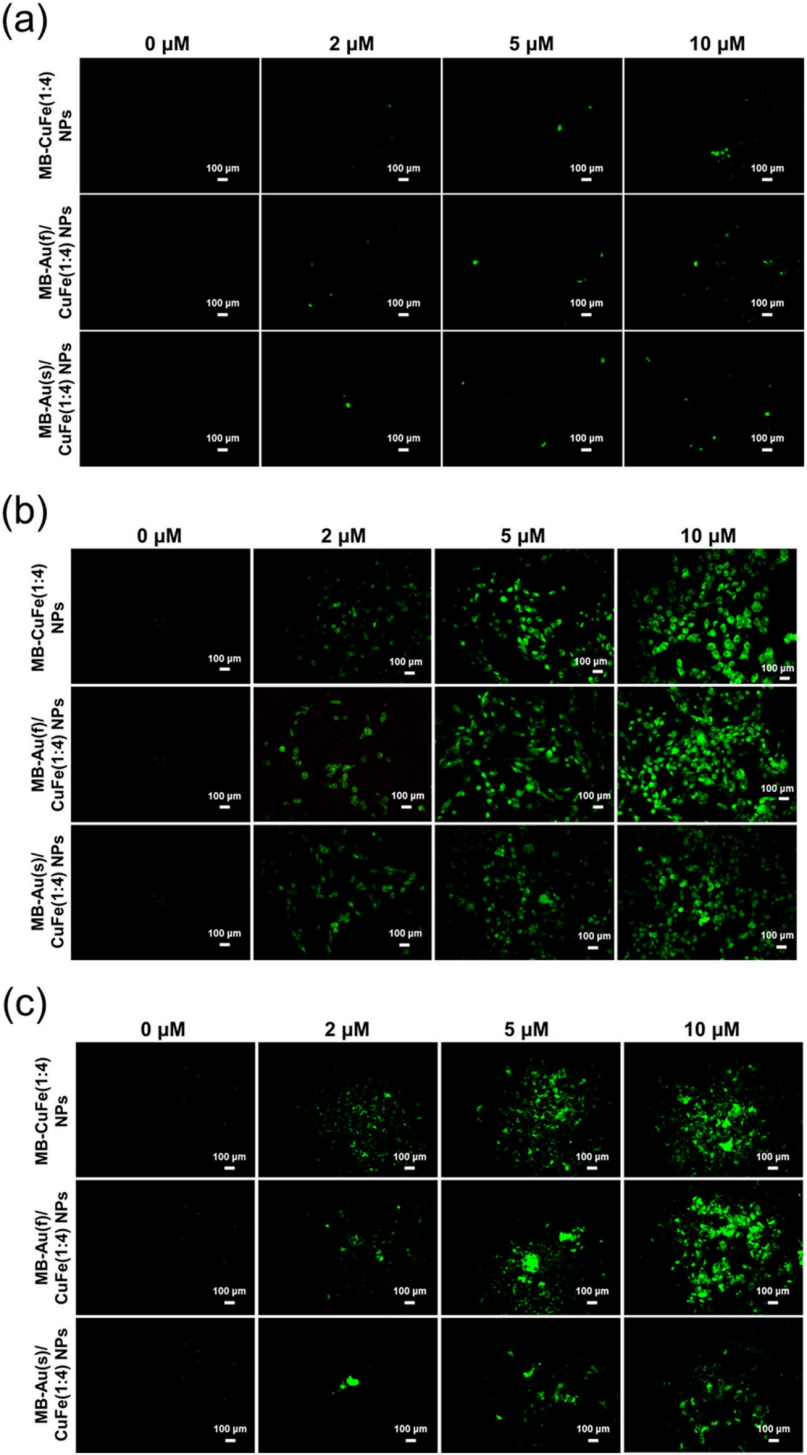
The DCFH-DA performance. (**a**) Without light irradiation. (**b**) With light irradiation after 24 h of incubation. (**c**) With light irradiation after 48 h of incubation.

The ROS generation at different MB concentrations and different incubation times was detected via DCFH-DA assay. The DCF fluorescence intensity was proportional to the concentration of ROS. In Fig. 6a, there is a weak detected signal even at the MB concentration of 10 uM for all groups before light irradiation. However, after light irradiation and another 24 h of incubation, the concentration of ROS is elevated with the increase of MB concentration. Among three groups, CuFe (1:4) NPs and Au(f)/CuFe NPs show the relatively more vigorous DCF fluorescence intensity, compared to Au(s)/CuFe NPs (Fig. 6b). With another 48 h of incubation after laser treatment, the DCF fluorescence could still be detected, which implies the chemodynamic effect continued working (Fig. 6c).

**Figure 7:**
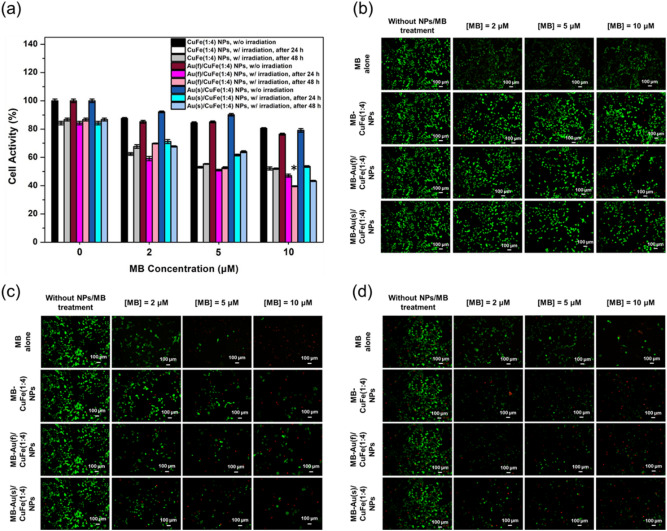
(**a**) Cell activity of NPs with and without light irradiation for 10 min after 24 h and 48 h incubation. (n = 6). (**b**) The Live/Dead assay of the groups without light irradiation. (**c**) The Live/Dead assay of the groups with light irradiation after 24 h. (**d**) The Live/Dead assay of the groups with light irradiation after 48 h.

Photoinduced toxicity was estimated by MTT assay and Live/Dead assay to understand the numerical and morphological presentation of cell activity before and after light irradiation. The photoinduced groups were tracked for a further 24 and 48 h (Fig. 7a). Without the help of a laser, each group showed weak toxicity resulting from a chemodynamic effect. In contrast, the toxicity increased after light irradiation, which was averagely a drop of 30% in cell activity contributed by the photodynamic effect. After the irradiation, the ROS-mediated effect still occurred, resulting in second-stage cell apoptosis led by a chemodynamic effect, especially for the groups with 10 uM of MB. With the light irradiation after 24 h, each group showed matchable toxicity. However, it could still be observed that the Au(f)/CuFe(1:4) NP had the highest toxicity, with the CuFe(1:4) NP the next, and the Au(s)/CuFe(1:4) NP the lowest as the previous result. However, with the elevation in doses and the passing of time, the toxicity of the Au(f)/CuFe(1:4) NP was gradually revealed, which showed the lowest cell activity at around 39% after 48 h of incubation. Compared to the CuFe(1:4) NP and Au(s)/CuFe(1:4) NP, it was determined to be the best material for performing the ROS-mediated effect.

The cell activity of HeLa cells could also be morphologically observed by Live/Dead assay. Before light treatment, there were more live cells than dead ones in each group. (Fig. 7b) After light treatment and another 24 h of incubation, the number of live cells decreased, and the dead cells increased with the elevation of doses of MB concentration (Fig. 7c). When the incubation time was stretched to 48 h, the amount of the live cells increased for the groups with low doses of NPs because of cellular differentiation. Nevertheless, for the groups with high doses of NPs, the amount of the dead cells continued increasing with the help of the second-stage chemodynamic effect (Fig. 7d).

## Conclusion

In this research, we successfully controlled the metal composition of Au/CuFe nanoparticles (NPs) by adjusting two factors: the Fe/Cu ratio of CuFe NPs and the addition of orders of the Au doping reactants. The characteristics of different Au/CuFe NPs were measured and quantified. The UV–visible spectrum showed the absorption peak at 500 ~ 600 nm; the Au(f)/CuFe NPs had a wide bandwidth, while the Au(s)/CuFe NPs had a narrow one. The shapes were round and with multiple cores. Au/CuFe NPs were also capable of finely dispersing in DI water with hydrodynamic sizes below 200 nm. The stability test showed that Au/CuFe NPs were stable in either acidic or neutral conditions, while the nanostructure changed within 4 h in a basic solution. It was also confirmed that the Au/CuFe NPs failed to trigger a photothermal effect when exposed to a 660 nm laser.

The result of the MTT assay indicated the low cytotoxicity of the Au/CuFe(4:1) NPs, compared with Au/CuFe(1:4) NPs. High doses of Au/CuFe(1:4) NPs were able to trigger cell apoptosis via a chemodynamic effect.

Finally, the ROS-mediated effect was examined. The methylene blue (MB)-immobilized Au/CuFe NPs were formed and checked by UV–visible spectrum and dynamic light scattering analysis. These NPs underwent RNO/imidazole assay and a TA test. MB-NPs with an equivalent amount of MB showed the matchable performance of generating singlet oxygen, which is around 75% compared with MB only, and hydroxyl radicals could be produced via Au/CuFe nanoreactors, which MB-Au(f)/CuFe(1:4) NPs showed the best chemodynamic effect as the concentration of MB was fixed. The dark and photoinduced toxicity were also done; Au (f)/CuFe(1:4) NPs showed the highest toxicity before and after light irradiation at a suitable dose with the help of chemodynamic and photodynamic synergic ablation.

In conclusion, we modified the structure of CuFe oxide-polymer nanoparticles with an additional Au doping reaction. The results showed Au/CuFe oxide-polymer nanoparticles synthesized with CuFe(1:4) oxide-polymer ones. This first Au doping process could present great ability of singlet oxygen and hydroxyl radical generation and effective in vitro ROS generation. We believe that the Au/CuFe oxide-polymer nanoparticles show potential as a chemodynamic/photodynamic synergetic therapy agent.

## Materials and methods

### Materials

Copper (II) chloride dehydrate (Riedel-de Haën, USA), Iron(II) chloride anhydrous(Alfa Aesar, USA), Hydrogen tetrachloroaurate(III) hydrate(Alfa Aesar, USA), (1-Hexadecyl) trimethylammonium bromide(Alfa Aesar, USA), Hydrazine hydrate(Acros Organics, USA), Poly(styrene-alt-maleic acid) sodium salt solution (PSMA)(Aldrich, USA), L-Ascorbic acid(Sigma-Aldrich, USA), Hydrogen peroxide solution(Sigma, USA), p-Toluenesulfonic Acid, 12% in Acetic Acid (TA) (Acros Organics, USA), N, N-Dimethyl-4-nitrosoaniline (RNO) (Alfa Aesar, USA).

### Au/CuFe oxide-polymer NPs synthesis

All the reactants in Table 2 were added to a 23 mL Teflon-lined hydrothermal synthesis autoclave reactor. Then the reactor was heated to 158 ℃ in an oven for 6 h, which is presented in Table 2. The reactor was then placed at room temperature for 1 h to cool down. The product solution was centrifuged at 12,000 rpm for 10 min to precipitate the nanoparticles. The supernatant was removed, and the precipitant was resuspended in DI water. The washing process was repeated three times. The product underwent low-speed centrifugation at 1800 rpm for 5 min to remove a significant accumulation of particles. The supernatant was preserved and diluted to 52 ppm of Cu with DI water for further experiments.

**Table 2:** The formula of CuFe oxide-polymer NPs synthesis.

Chemical/Parameter	Concentration	Fe/Cu ratio of reactant	
		0.25	4
PSMA	240 mg/mL	2.5 mL	2.5 mL
DI water	–	7.375 mL	5.5 mL
CuCl_2_	5 mM	1 mL	1 mL
FeCl_2_	10 mM	0.125 mL	2 mL
HCl	2 M	18 μL	18 μL
N_2_H_4_	64%	100 μL	100 μL
Heating time	–	6 h	6 h

CuFe oxide-polymer NP products with different Fe/Cu ratios were used in the Au/CuFe oxide-polymer NP synthesis. All the reactants needed are shown in Table 3. CTAB and HAuCl_4_ were first mixed in a glass tube for 5 min, forming an orange-yellow complex. Then Vitamin C and CuFe oxide-polymer were added following different orders of addition for a total reaction time of 25 min to form the first and second products (Au(f)/CuFe NPs and Au(s)/CuFe NPs with f: first and s: second). After 30 min cooling, the product solution was centrifuged at 12,000 rpm for 10 min to precipitate the nanoparticles. The supernatant was removed, and the precipitant was resuspended in DI water. The washing process was repeated three times. The supernatant was preserved and filled up to 500 μL with DI water for further experiments.

**Table 3:** The formula of Au/CuFe oxide-polymer NPs synthesis.

Chemical/Parameter	Concentration	Volume of reactant
CTAB	52 mg/mL	1750 μL
HAuCl_4_	5 mM	250 μL
Vitamin C	17.6 mg/mL	500 μL
CuFe oxide-polymer NPs	52 ppm Cu	500 μL
Heating time	–	20 In

### Quantification of copper, iron, and gold

The metal core and polymer had to be broken down to quantify the metal concentration. One hundred μL of NP solution were first mixed with 225 μL of 12 M HCl and 225 μL of 16 M HNO_3_ to dissolve metal, then mixed with 1800 μL of 4.5 M NaOH to destroy PSMA polymer. Finally, 300 μL of 12 M HCl and 1350 μL of DI water were added to adjust the solution to the acidic condition. Then the concentration of copper, iron, and gold was quantified separately by an atomic absorption analyzer.

### Metal ratio and optical properties

The metal ratio was quantified by an atomic absorption analyzer. The optical properties of Au/CuFe NPs before and after HCl corrosion with and without magnetic separation were characterized by UV–visible spectroscopy from the wavelength of 800 to 400 nm and the scanning speed was 10 nm/sec. To confirm that AuNPs were successfully doped in the core of the nanostructure, Au/CuFe NPs were etched by 0.05 M HCl for 20 min, followed by centrifugation at 12000 rpm for 10 min. They were resuspended in DI water for further measurement.

### Structures, size distribution, and zeta potential

Transmission electron microscopy was applied to determine the structure of Au/CuFe NPs before and after HCl corrosion. The size distribution and zeta potential were determined by dynamic light scattering. An X-ray diffractometer (Malvern Panalytical) was used to determine Powder diffraction analysis.

### Stability test

Au/CuFe NPs were dispersed in acidic PBS (pH = 4.0), neutral PBS (pH = 7.4) and basic PBS (pH = 10.0) then placed at room temperature. At different time intervals, the optical properties of Au/CuFe NPs solutions were measured by UV–visible UV–visible spectroscopy.

### Cell culture

In this research, HeLa cells were applied in all in vitro experiments, and the cells were obtained from the Department of Plant Pathology and Microbiology, National Taiwan University. The cells were cultured in a DMEM-HG culture medium with 10% FBS in a 10-cm culture dish and placed in an incubator at 37 ℃ and 5% CO_2_. After appropriate proliferation time, which was usually 1 or 2 days, the cells covered 70% of the culture dish. After being washed by PBS once, trypsin–EDTA was added to detach the cells. The detachment process took 4 min at 37 ℃. 5 mL of culture medium was added to inhibit trypsin activity, and the cells were transferred to a 15-mL centrifuge tube. After 900 rpm centrifugation for 5 min at 4 ℃, the supernatant was removed, an appropriate amount of culture medium was added, and the cells were resuspended. To estimate the concentration of cells, 20 μL of trypan blue was mixed with 20 μL of suspended cell solution. A hemocytometer was used to determine the concentration of viable cells.

### Cytotoxicity of Au/CuFe NPs

First, the cells were diluted to 50000 cells/mL with a culture medium. One hundred μL of cells were added to a 96-well plate for 24 h. Then the cells were washed by PBS once, and 100 μL of culture medium with different metal concentrations of Au/CuFe NPs suspended were added. After 24 h incubation, the culture medium containing NPs was removed, and the cells were washed with PBS once. The MTT working stock was diluted by 10 folds with a 0.5 mg/mL culture medium. 100 μL culture medium containing MTT was added, and the cells then reacted with MTT for 3.5 h in the incubator. The medium was removed, and 100 μL DMSO was added to dissolve formazan produced by viable cells. After shaking for 30 min avoiding light, the absorbance at 570 nm was measured by a microplate reader. The blank absorbance was the well with no cells but treated with MTT culture medium for 3.5 h.

and then replaced by 100 μL DMSO. The cell activity was defined as the ratio between the NP-treated and non-treated groups.

### Methylene blue loading and purification

CuFe NPs and Au/CuFe NPs with 150 ppm of Fe were immobilized with 0.25 mM methylene blue (MB) under agitation. After 18 h loading, the solution was centrifuged at 11,000 rpm for 10 min to precipitate the MB-loaded CuFe NPs and Au/CuFe NPs (MB-CuFe NPs and MB-Au/CuFe NPs). The precipitant was resuspended in DI water for further use while the supernatant was collected, followed by another centrifugation at 13,000 rpm for 10 min. The washing process was repeated three times. The supernatant was collected and measured by the UV–visible spectrometer. The calibration line was established by the absorbance at 660 nm of different MB concentrations. The unloaded MB in the supernatant was quantified, which could be used to calculate the amount of immobilized MB. Furthermore, the encapsulation efficiency (EE%) can be determined by the following formula,

$$EE\left(\%\right)=\frac{{N}_{t}-{N}_{f}}{{N}_{t}}\times 100\%$$

N_t_ is the amount of total MB added, and N_f_ is that of free non-entrapped MB. The loading capacity (LC%) can also be calculated by the following formula,

$$LD\left(\%\right)=\frac{{N}_{MB}}{{N}_{NP}}\times 100\%$$

where N_MB_ is the amount of encapsulated MB, and N_NP_ is that of metal NPs.

### Ability of singlet oxygen generation

To understand if MB-Au/CuFe NPs could generate singlet oxygen (^1^O_2_) under irradiation, N, N-Dimethyl-4-nitrosoaniline (RNO), and imidazole were applied in this assay. After reacting with ^1^O_2_, the bleaching of RNO could be observed. 1 mL of a solution that consisted of 10 μM of MB, 0.025 mM of RNO, and 0.2 mM of imidazole were placed in a 1.5-mL Eppendorf tube. Each group was irradiated with a 660 nm laser at 75 mW/cm^2^ for 10 min. The reducing absorbance at 440 nm was observed and quantified via UV–visible spectroscopy. The UV–visible slit was switched at the wavelength of 350 nm.

### Ability of hydroxyl radical generation

To understand if Au/CuFe NPs and MB-Au/CuFe NPs could catalyze H_2_O_2_ degradation and accelerate hydroxyl radicals (·OH) generation, p-Toluenesulfonic Acid (TA) was applied. After reacting with ·OH, TA turned into 2-hydroxyterephthalic acid (TAOH), a fluorescent agent. In this assay, the Cu, Fe, Au, and MB concentrations were fixed at 1 ppm, 20 ppm, 60 ppm, and 10 uM, respectively. 100 μL of solution that consisted of a corresponding concentration of Au/CuFe NPs or MB-Au/CuFe NPs, 5 mM of TA, and 500 μM of H_2_O_2_ were plated in a 96-well plate and reacted for 24 h. The fluorescence intensity was quantified by a multi-mode microplate reader at the excitation wavelength of 310 nm and the emission wavelength of 426 nm.

### Cellular uptake

The cellular uptake was determined by quantifying the MB endocytosed via UV–visible spectroscopy. The cells were first diluted to 50000 cells/mL with a culture medium, and 100 μL of cells were added to 96-well plate for 24 h. Then the cells were washed by PBS once, and 100 μL of culture medium containing 10 μM MB concentration of MB-CuFe NPs or MB-Au/CuFe NPs was added. After 4 h incubation with and without a magnetic field, the medium was removed. Each well was washed by PBS twice, and 100 μL of DMSO was added for the following measurements. The corresponding cell activity was determined by MTT assay.

### Dark toxicity

The cells were diluted to 50000 cells/mL with culture medium. One hundred μL of cells was added to a 96-well plate for 24 h. Then the cells were washed by PBS once, and 100 μL of culture medium with different MB concentrations of MB-CuFe NPs or MB-Au/CuFe NPs suspended in were added. After 24 h incubation, the cell activity was determined by MTT assay.

### Detection of in-vitro reactive oxygen species generation

To understand if MB-Au/CuFe NPs could boost in vitro reactive oxygen species (ROS) generation under irradiation, 2′, 7′-Dichlorofluorescin diacetate (DCFH-DA) was applied in this assay. After reacting with ROS, DCFH-DA turned into 2′-7′-dichlorofluorescein (DCF), a fluorescent agent. HeLa cells were seeded into a 24-well plate at a density of 12,000 cells/well and incubated for 24 h. After removing the medium, the cells were washed with PBS once. One mL of medium that consisted of MB-CuFe NPs or MB-Au/CuFe NPs with different concentrations of MB were added and co-cultured with the cells for 24 and 48 h, respectively. After removing the medium, the cells were washed with PBS once. The DCFH-DA stock solution was diluted with culture medium to 20 μM and added to each well. After incubating for 30 min, each well was irradiated with a 660 nm laser at 75 mW/cm^2^ for 10 min, followed by another 60 min incubation. After removing the solution, the cells were washed with PBS once. The fluorescence exhibited was observed with an inverted fluorescence microscope.

### Photoinduced toxicity

HeLa cells were seeded into a 24-well plate at a density of 12,000 cells/well and incubated for 24 h. After removing the medium, the cells were washed with PBS once. One mL of medium that consisted of MB-CuFe NPs or MB-Au/CuFe NPs with different concentrations of MB was added. After incubating with the cells for 4 h, each well was irradiated with a 660 nm laser at 75 mW/cm^2^ for 10 min, followed by another 24 h and 48 h incubation. The cell activity was examined by MTT assay and observed by Live/Dead assay.

### Statistical analysis

The experimental data were shown as average value ± standard deviation (SD). One-way ANOVA for multiple group comparisons was applied to estimate whether existed statistically significant data (difference), counting *P* > *0.05*, non-significant (n.s.).

## Supplementary Information

Supplementary Information.

## Data Availability

The datasets generated during and/or analyzed during the current study are available from the corresponding author on request.
