# Supplementary material for: Fabrication of an Au-doped Cu/Fe oxide-polymer core–shell nanoreactor with chemodynamic and photodynamic dual effects as potential cancer therapeutic agents
Source: Sci Rep. 2022 Nov 4;12:18729. doi: 10.1038/s41598-022-23002-5 (PMC9636373; doi:10.1038/s41598-022-23002-5)
Supplement: Supplementary file 1 — Supplementary Information. [file 41598_2022_23002_MOESM1_ESM.docx]

Supporting information

Fabrication of Au-doped Cu/Fe Oxide-polymer Core-shell Nanoreactor with Chemodynamic and Photodynamic Dual Effects as Potential Cancer Therapeutics Agents

Chun-Kai Sun^1^, Yin-Hsu Wang^1^_,_Yu-Liang Chen^1^, Ting-Yu Lu^2^, Hsi-Ying Chen^4^, Shih-Chin Pan^3^,Po-Chun Chen^3^, Mei-Yi Liao^4^* and Jiashing Yu^1^*

*^1^ Department of Chemical Engineering, National Taiwan University, Taipei 10617, Taiwan*

*^2^ Materials Science and Engineering Program, University of California San Diego, La Jolla, CA, 92093, USA*

*^3^ Department of Materials and Mineral Resources Engineering, Institute of Materials Science and Engineering, National Taipei University of Technology, Taipei 10608, Taiwan*

*^4^ Department of Applied Chemistry, National Pingtung University, Pingtung 90003, Taiwan*

*Corresponding authors:

Mei-Yi Liao, Department of Applied Chemistry, National Pingtung University, Pingtung 90003, Taiwan, E-mail: myliao@mail.nptu.edu.tw

Jiashing Yu, Department of Chemical Engineering, National Taiwan University, Taipei 10617, Taiwan, E-mail: [jiayu@ntu.edu.tw](mailto:jiayu@ntu.edu.tw)


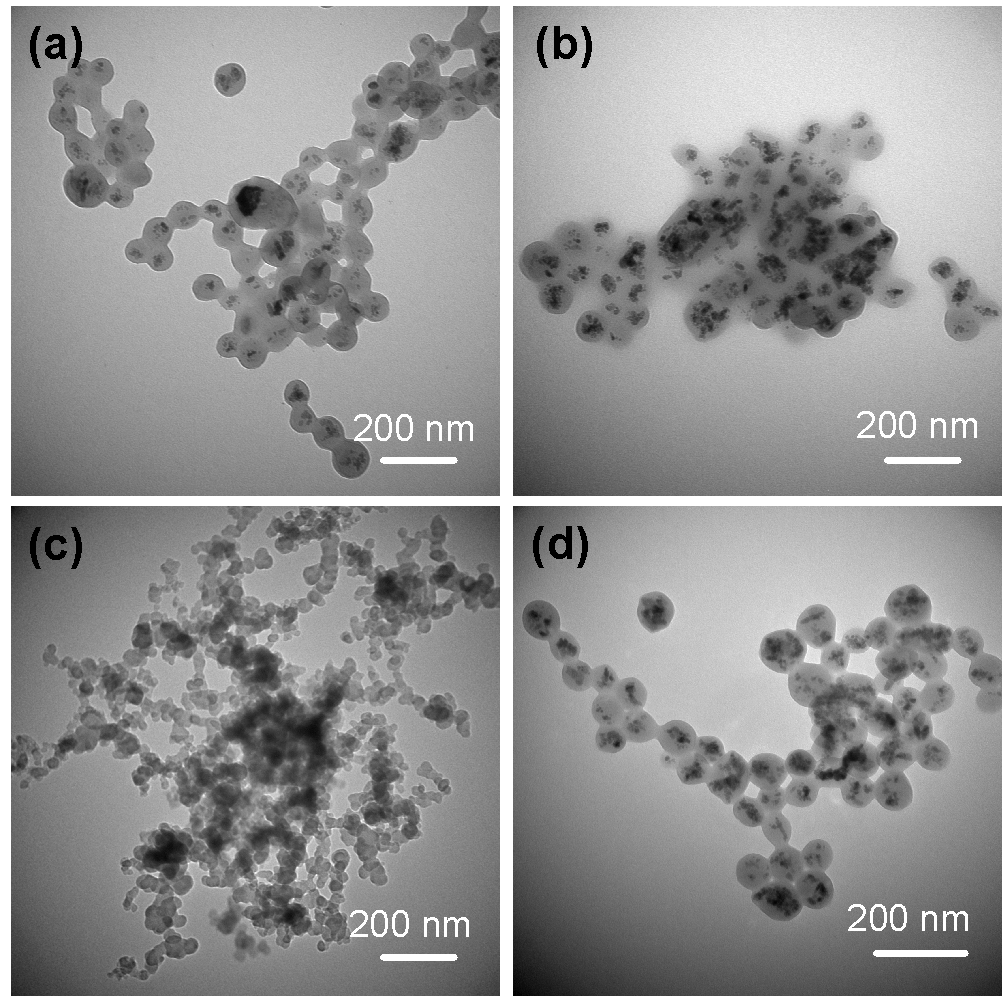


**Figure S1**. TEM images (a-d) after HCl corrosion. (a) Au(f)/CuFe(4:1) NPs. (b) Au(s)/CuFe(4:1) NPs. (c) Au(f)/CuFe(1:4) NPs. (d) Au(s)/CuFe(1:4) NPs. Scale bar: 100 nm.


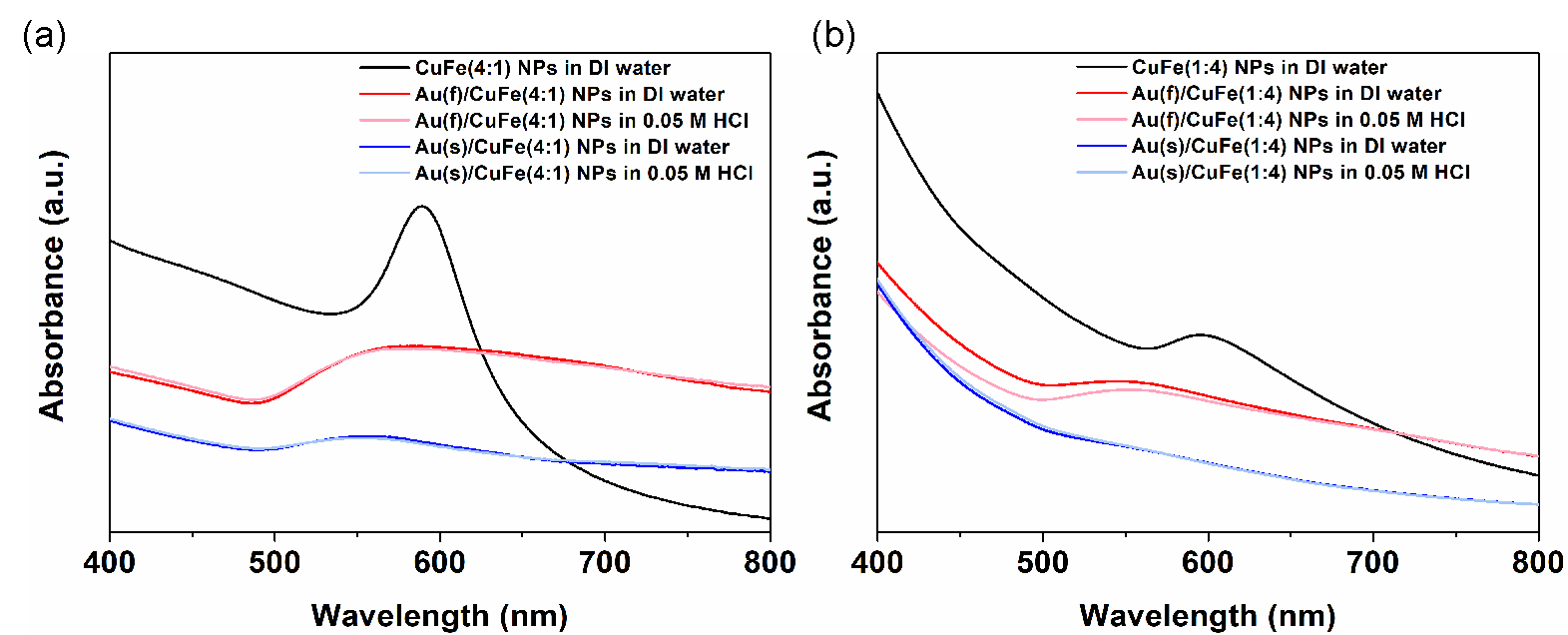


**Figure S2** UV-visible spectrum of (a) Au/CuFe(4:1) NPs and (b) Au/CuFe(1:4) NPs before and after 0.05 M HCl corrosion.

To characterize the behavior of Au/CuFe NPs in the aqueous phase, dynamic light scattering (DLS) was applied to determine the hydrodynamic diameter and zeta potential. In **Figure S3 (a)**, Au(s)/CuFe(4:1) NP and Au(s)/CuFe(1:4) NP both show a larger size than Au(f)/CuFe(4:1) NP and Au(f)/CuFe(1:4) NP; the phenomenon can also be observed in TEM images. In **Figure S3 (b)**, after the Au doping reaction, the surface charge changes from negative to positive due to the presence of CTAB at the shell of Au/CuFe NPs, and the first process leads to more positively-charged NPs than the second one.


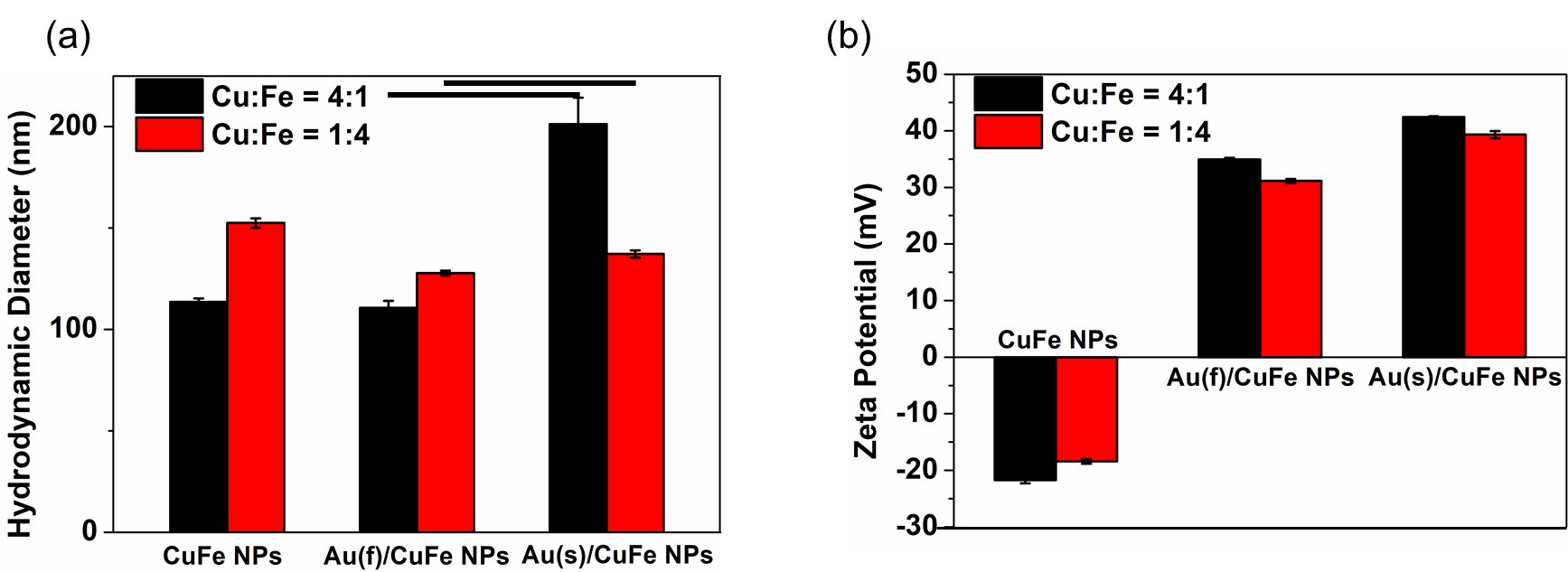


**Figure S3**. Dynamic light scattering (DLS) analysis. (a) Hydrodynamic diameter and (b) zeta potential of CuFe NPs and Au/CuFe NPs. (n = 3)

**Stability Test**

To understand the stability of NPs, different Au/CuFe NPs were dispersed in PBS solvent with three different pH values, including 4.0 (acidic), 7.4 (neutral), and 10.0 (basic). The solution was centrifuged at different time intervals, and optical properties were measured. (**Figure S4**) In the PBS- NPs system, the optical change can be ascribed to salt-induced aggregation. For each group of Au/CuFe NPs, the gradual decrease of the absorption peak in intensity can be observed in pH 4.0 and 7.4 PBS; however, in pH 10.0 PBS, the absorption peaks are fast weakened after 4 h quiescence.


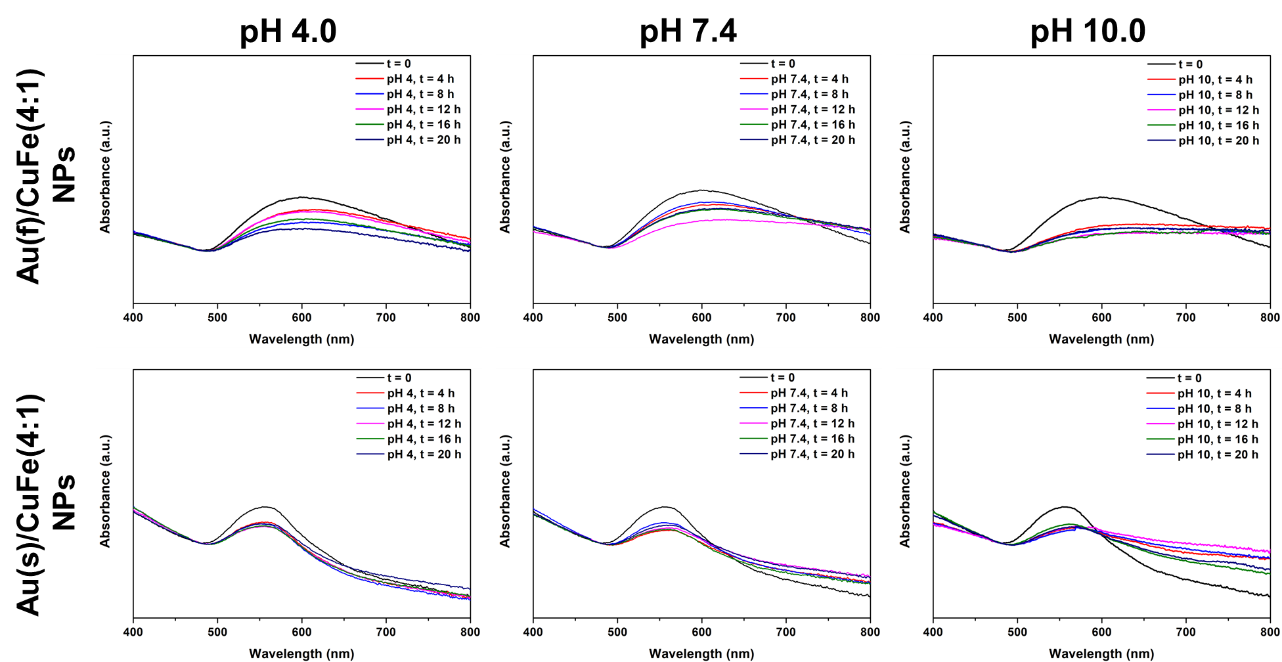


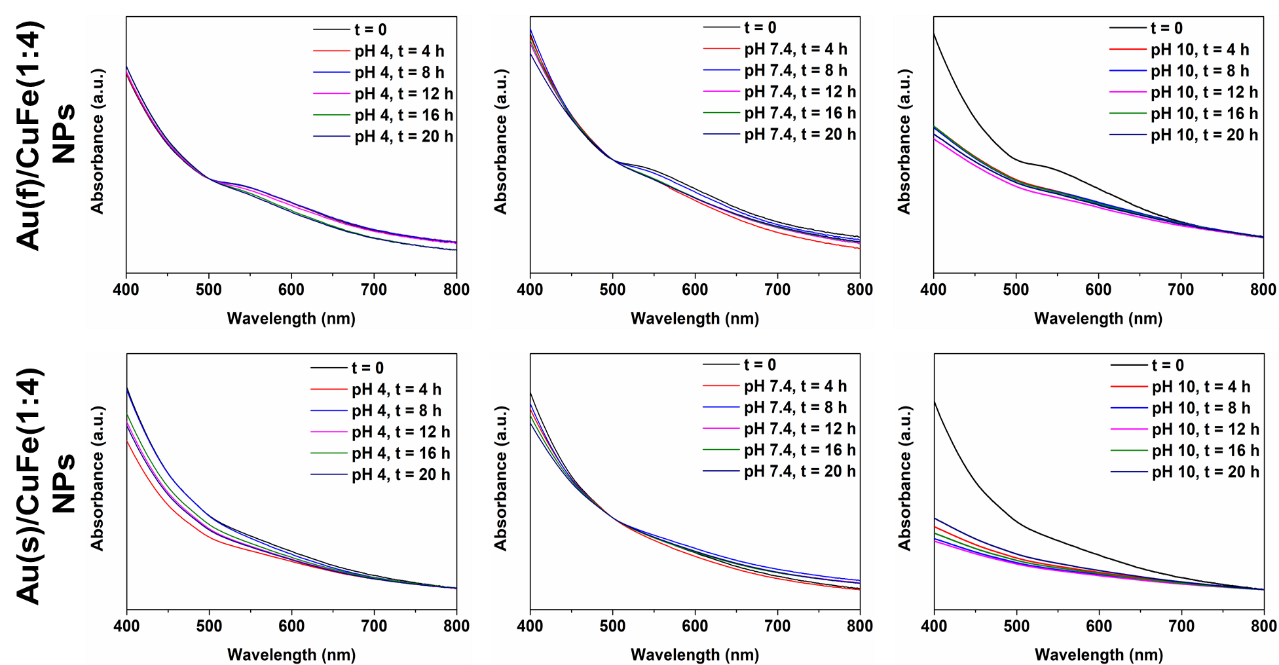


**Figure S4.** Optical properties of Au/CuFe NPs after 20 h dispersion in PBS at 25 ℃.


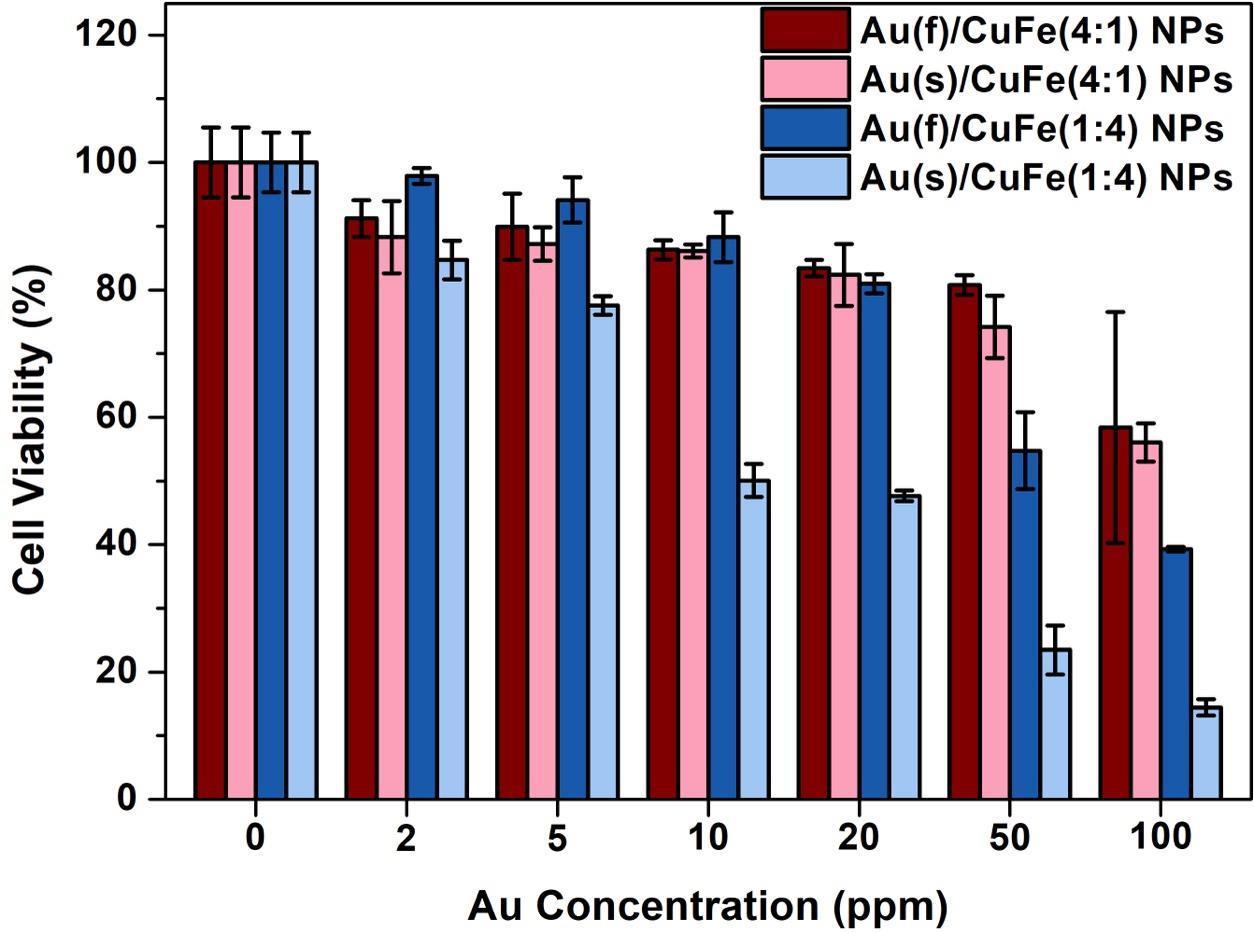


**Figure S5**. Cell viability of Au/CuFe NPs after 24 h incubation. (n = 4)


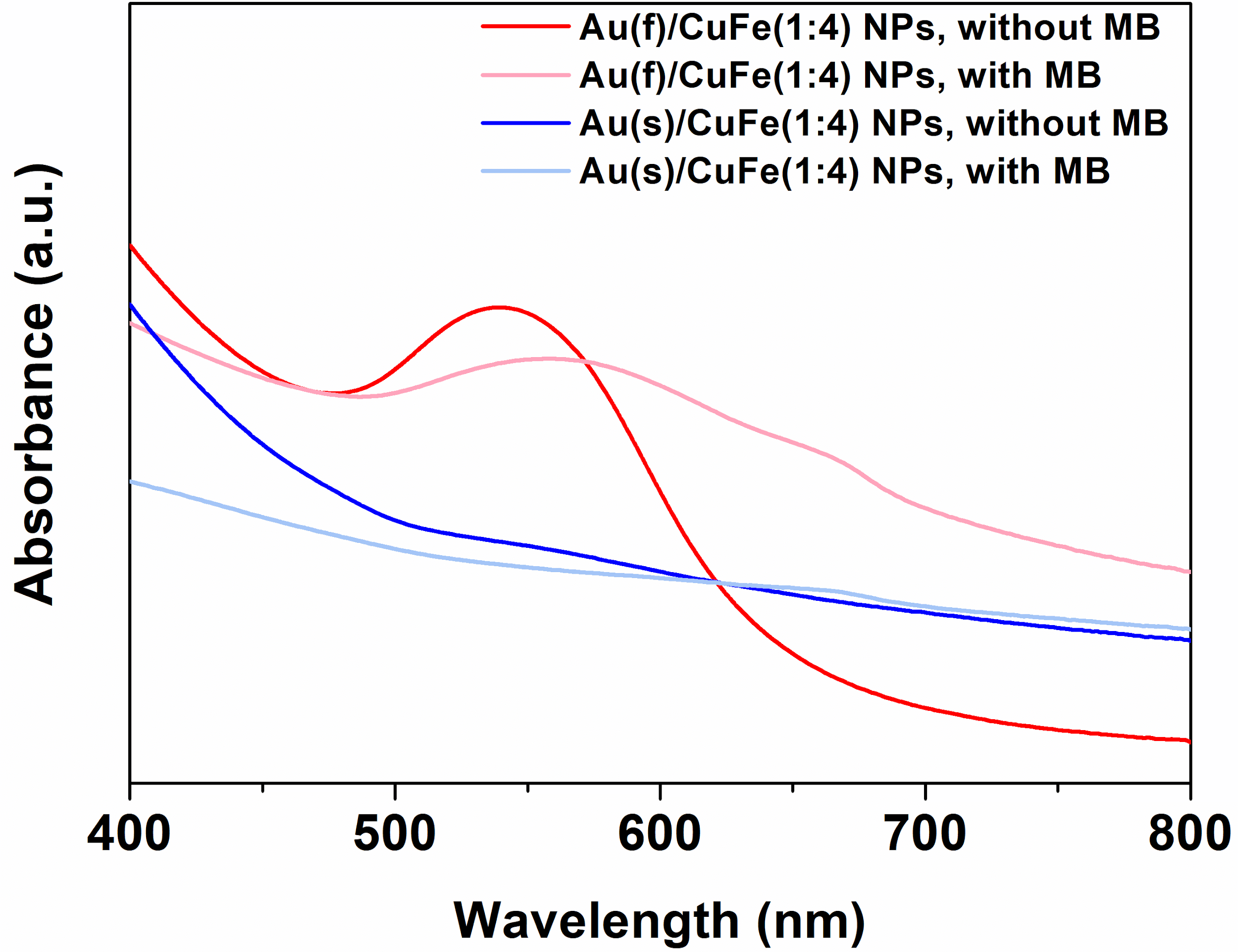


**Figure S6.** UV-Vis spectra of Au(f)/CuFe(4:1) NPs and Au(s)/CuFe(4:1) NPs loaded with methylene blue (MB).

**Table S1**. Composition of Au/CuFe(1:4) oxide-polymer core-shell NPs with 10 μM of MB. (n = 3)

| Types of metal oxide-polymer MB-NPs | Concentration | | |
| --- | --- | --- | --- |
|  | Cu (ppm) | Fe (ppm) | Au (ppm) |
| CuFe(1:4) | 3.14 $\pm$ 0.01 | 15.46 $\pm$ 0.07 | - |
| Au(f)/CuFe(1:4) | 1.38 $\pm$ 0.06 | 22.56 $\pm$ 0.95 | 82.93 $\pm$ 3.49 |
| Au(s)/CuFe(1:4) | 0.63 $\pm$ 0.01 | 24.11 $\pm$ 0.11 | - 1. $\pm$ 0.33 |


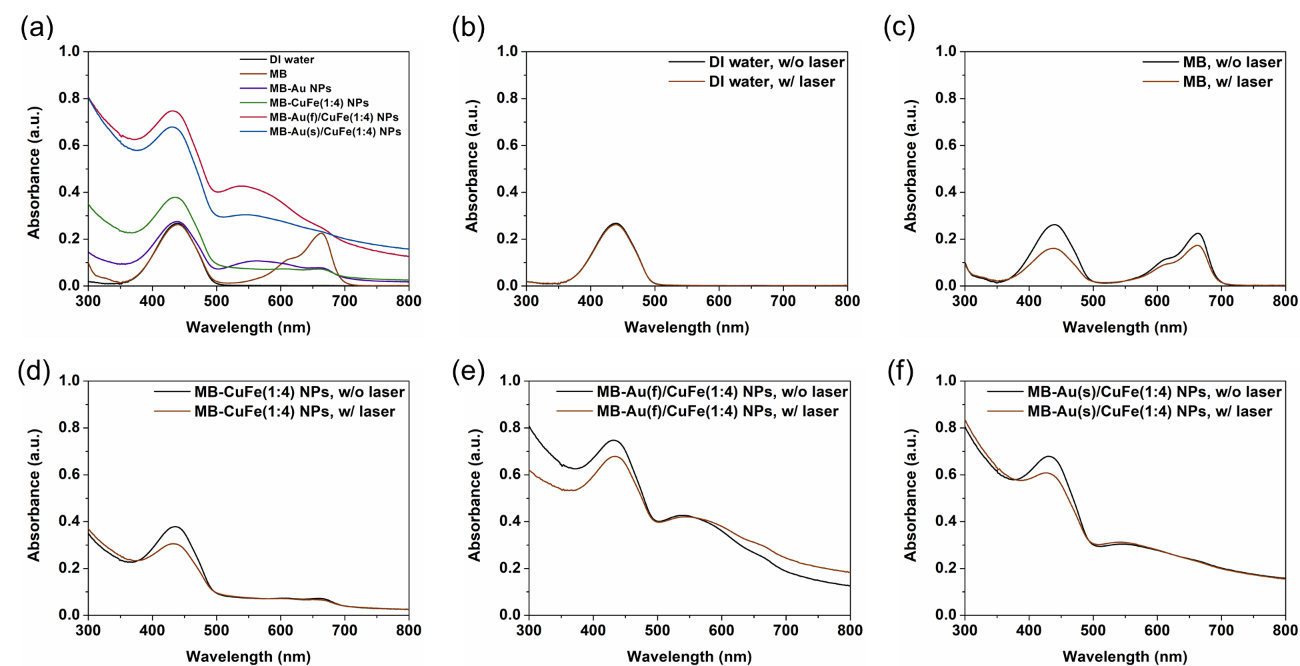


**Figure S8**. The abilityinglet oxygen generation. (a) The RNO-contributed characteristic peak at 440 nm. (b) DI water, (c) MB, (d) MB-CuFe(1:4) NPs, (e) MB-Au(f)/CuFe(1:4) NPs and (f) MB-Au(s)/CuFe(1:4) NPs after 10 min of light irradiation.

**Table S2**. Ability of singlet oxygen generation compared to MB only.

| DI water | MB | CuFe NPs | Au(f)/CuFe NPs | Au(s)/CuFe NPs |
| --- | --- | --- | --- | --- |
| 0.00 % | 100.00 % | 79.55 % | 73.48 % |  |

**Figure S9**. The dissolution experiment of Cu and Fe under various culture conditions.


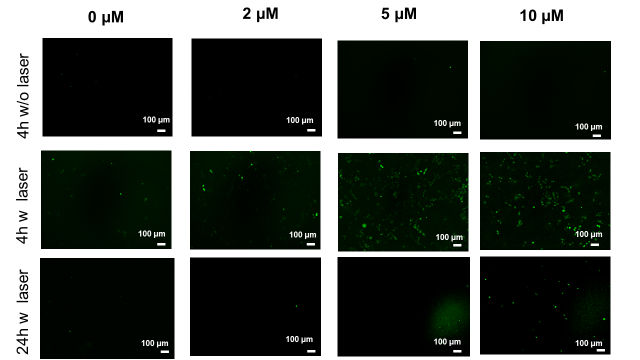


**Figure S10**. The DCFH-DA performance of MB only without light irradiation, with light irradiation after 4h and 24h of incubation.
